# *BDNF* Val66Met Polymorphism Is Associated With Motor Recovery After Rehabilitation in Progressive Multiple Sclerosis Patients

**DOI:** 10.3389/fneur.2022.790360

**Published:** 2022-02-21

**Authors:** Antonino Giordano, Ferdinando Clarelli, Miryam Cannizzaro, Elisabetta Mascia, Silvia Santoro, Melissa Sorosina, Laura Ferrè, Letizia Leocani, Federica Esposito

**Affiliations:** ^1^Laboratory of Human Genetics of Neurological Disorders, Institute of Experimental Neurology (INSPE), IRCCS San Raffaele Scientific Institute, Milan, Italy; ^2^Neurology Unit, IRCCS San Raffaele Scientific Institute, Milan, Italy; ^3^Vita-Salute San Raffaele University, Milan, Italy; ^4^Neurorehabilitation Unit, IRCCS San Raffaele Scientific Institute, Milan, Italy; ^5^Experimental Neurophysiology Unit, Institute of Experimental Neurology (INSPE), IRCCS San Raffaele Scientific Institute, Milan, Italy

**Keywords:** *BDNF*, multiple sclerosis, rehabilitation, progressive, motor recovery, neurotrophin, biomarker

## Abstract

**Background:**

Rehabilitation is fundamental for progressive multiple sclerosis (MS), but predictive biomarkers of motor recovery are lacking, making patient selection difficult. Motor recovery depends on synaptic plasticity, in which the Brain-Derived Neurotrophic Factor (BDNF) is a key player, through its binding to the Neurotrophic-Tyrosine Kinase-2 (NTRK2) receptor. Therefore, genetic polymorphisms in the BDNF pathway may impact motor recovery. The most well-known polymorphism in *BDNF* gene (rs6265) causes valine to methionine substitution (Val66Met) and it influences memory and motor learning in healthy individuals and neurodegenerative diseases. To date, no studies have explored whether polymorphisms in *BDNF* or *NTRK2* genes may impact motor recovery in MS.

**Objectives:**

To assess whether genetic variants in *BDNF* and *NTRK2* genes affect motor recovery after rehabilitation in progressive MS.

**Methods:**

The association between motor recovery after intensive neurorehabilitation and polymorphisms in *BDNF* (rs6265) and *NTKR2* receptor (rs2289656 and rs1212171) was assessed using Six-Minutes-Walking-Test (6MWT), 10-Metres-Test (10MT) and Nine-Hole-Peg-Test (9HPT) in 100 progressive MS patients.

**Results:**

We observed greater improvement at 6MWT after rehabilitation in carriers of the *BDNF* Val66Met substitution, compared to *BDNF* Val homozygotes (*p* = 0.024). No significant association was found for 10MT and 9HPT. *NTRK2* polymorphisms did not affect the results of motor function tests.

**Conclusion:**

*BDNF* Val66Met was associated with walking function improvement after rehabilitation in progressive MS patients. This result is in line with previous evidence showing a protective effect of Val66Met substitution on brain atrophy in MS. Larger studies are needed to explore its potential as a predictive biomarker of rehabilitation outcome.

## Introduction

Multiple sclerosis (MS) is a complex chronic neurological disorder characterized by inflammation, axonal degeneration, and demyelination. MS patients may present at onset with a relapse-remitting (RR) course, which can evolve into a secondary progressive (SP) course over the years, or with a primary progressive (PP) course. Despite the availability of many treatments to limit disease activity in RRMS, the pharmacological options for progressive MS are still lacking and the care of patients frequently involves physical therapy and rehabilitation (1, 2). The selection of MS patients who could benefit more from such interventions is often difficult and the mechanisms by which rehabilitation may reduce patients' disability in MS are not clear, but brain plasticity seems to have a determinant role (2).

One of the most important players involved in the biological pathways of brain plasticity is the Brain-Derived Neurotrophic Factor (BDNF). BDNF is a member of the neurotrophins family, and it binds to its specific receptor, the Neurotrophic Receptor Tyrosine Kinase 2 (NTRK2). The binding results in the activation of the MAPK pathway (3) and regulation of synaptic plasticity and repair. For this reason, BDNF has a critical role in different processes, including the development and differentiation of the central nervous system, long-term potentiation, memory formation and cognition (4–6).

A single nucleotide polymorphism (SNP) in the *BDNF* gene (rs6265), causing valine to methionine substitution (Val66Met), is known to lead to reduced production of BDNF (7). The Val66Met substitution plays a role, generally detrimental, in motor learning, cognition and memory in healthy individuals (8, 9) as well as in the severity of cognitive impairment, motor skills and recovery after neurological damage (10–13).

In the MS field, a few works focused on the role of the Val66Met polymorphism in influencing MS risk and disease activity (14), regional brain volumes (15–19) and memory functions (20). Given the biological relevance of the BDNF-dependent pathways in synaptic plasticity and repair, it is likely that genetic variants of these genes may have a role in motor recovery. However, no studies to date have explored whether *BDNF* Val66Met or other polymorphisms in its specific receptor may be associated with motor recovery after rehabilitation in MS.

The aim of the present study is to better investigate this hypothesis, testing the association of the *BDNF* Val66Met polymorphism (rs6265) and of two known polymorphisms in BDNF receptor (rs2289656 and rs1212171) with motor recovery after an intensive in-patient neurorehabilitation protocol in patients with progressive MS. The answer to this question could be important for the definition of predictors of motor recovery after rehabilitation, which could help in the selection of patients who could benefit more from such intervention.

## Methods

### Inclusion and Exclusion Criteria

We revised the medical records of patients admitted to the Neurorehabilitation Unit of San Raffaele Hospital (OSR) from January 2011 to December 2018 and applied the following inclusion and exclusion criteria.

Inclusion criteria: (a) diagnosis of PP or SP MS according to McDonald criteria (21); (b) availability of genotype data on the studied variants (see section SNPs Selection and Genotyping); (c) availability of clinical data regarding the results of at least one test between the chosen motor function tests (see section Motor Function Testing and Rehabilitation Program) both at admission and at discharge from the Neurorehabilitation Unit.

Exclusion criteria: (a) known relapses or evidence of inflammatory disease activity at MRI (e.g. new T2 lesions and/or gadolinium-enhancing lesions) within six months prior to the inclusion in the study; (b) history of any other disease that could affect motor abilities.

### Motor Function Testing and Rehabilitation Program

To assess patients' motor skills, we collected the results of the Six-minutes walking test (6MWT) (22), the Ten-Metres test (10MT) and the Nine-Hole Peg test (9HPT) (23), that had been routinely performed at admission and at discharge from the clinic. The 6MWT is widely accepted as a reliable tool to assess walk endurance and speed in MS. In this test, the patient is asked to walk at the fastest but safe possible speed for 6-min back and forth a 10-m hallway, turning around a line at each end. Every two min the patient is informed about elapsed time, with no further encouragement. The patient may halt to rest for a maximum time of 3 s, if needed; total distance walked is registered as result of the test. The 10MT is used to measure gait abilities in a short-distance performance. The patient is asked to walk for 10 m at maximum but safe speed; two different trials are performed, and the mean value is calculated. Finally, the 9HPT is used to assess manual dexterity. In this test, the subject is asked to repeatedly place and then remove nine pegs into nine holes, one at a time, as quickly as possible; total time for each hand is recorded.

All patients underwent an intensive inpatient neurorehabilitation program, consisting of daily physical therapy, performed twice a day for 5 days plus once on the 6th-day of the week, consecutively for three to four weeks. The rehabilitation program was tailored to patients' individual needs and encompassed passive stretching, flexibility, strengthening and balance exercises, and gait training.

### SNPs Selection and Genotyping

We focused our attention on relevant genetic variants that could have a high impact on BDNF function. For this reason, we selected 3 SNPs, one on *BDNF* gene and 2 on *NTRK2*. Specifically, for *BDNF* gene, the variant rs6265 (also known as Val66Met polymorphism) was chosen due to its known functionally relevant effects on BDNF and given the previous evidence showing its involvement in neurological diseases (7–13).

As regards the *NTRK2* gene, after a comprehensive research in PubMed and annotation databases [SNPedia: http://www.snpedia.com], we identified 20 known SNPs mapping to this gene, but for none of them a high impact such as the *BDNF* rs6265 was reported in literature. For this reason, we performed a functional impact prediction according to the GWAVA tool, which can predict the impact of non-coding genetic variants based on a wide range of annotations (24). Rs1212171 ranked first among the other *NTRK2* SNPs, showing the highest probability of having relevant functional effects. Additionally, we included in the study also rs2289656, which was already associated with major depression, suicide attempt risk and sporadic Alzheimer's disease (25–27).

In our cohort, whole genome genotyping data have been obtained through different Illumina® platforms (Infinium OmniExpress-24, HumanOmniExpress-12, Infinium Omni2.5-8 and Human660-Quad chip). All the patients had previously given their informed consent to the use of their genetic data for research purpose. Quality controls at whole genome-level were performed at SNP and individual level as previously described (28) using PLINK software, version 1.07 (29). From this dataset, the genotyping information of rs6265, rs121217 and rs2289656 were retrieved and used for the purpose of this study.

### Statistical Analysis

We used ANCOVA models to test the association between the selected SNPs and the change of the scores of the motor function tests, expressed as ratio values, in a pre-test/post-test design, before and after rehabilitation. We adjusted such analysis for the scores of the tests at baseline. Given the relatively small sample size, we focused on a dominant model, dividing patients in two groups according to their *BDNF* genotype (GG vs. AG/AA subjects, known also as Met-carriers). This approach was adopted also when analyzing the effect of the *NTRK2* gene variants (rs2289656: most common GG vs. AG/AA; rs1212171: most common TT vs. TC/CC). A sensitivity analysis did not reveal any significant impact of other factors (like age, gender, duration of the progressive phase) on the reported results, when included in the models. Statistical analyses were performed using R version 3.6.1, R Core Team, 2013 (30).

## Results

### Characteristics of Included MS Subjects at Baseline

**Figure 1:**
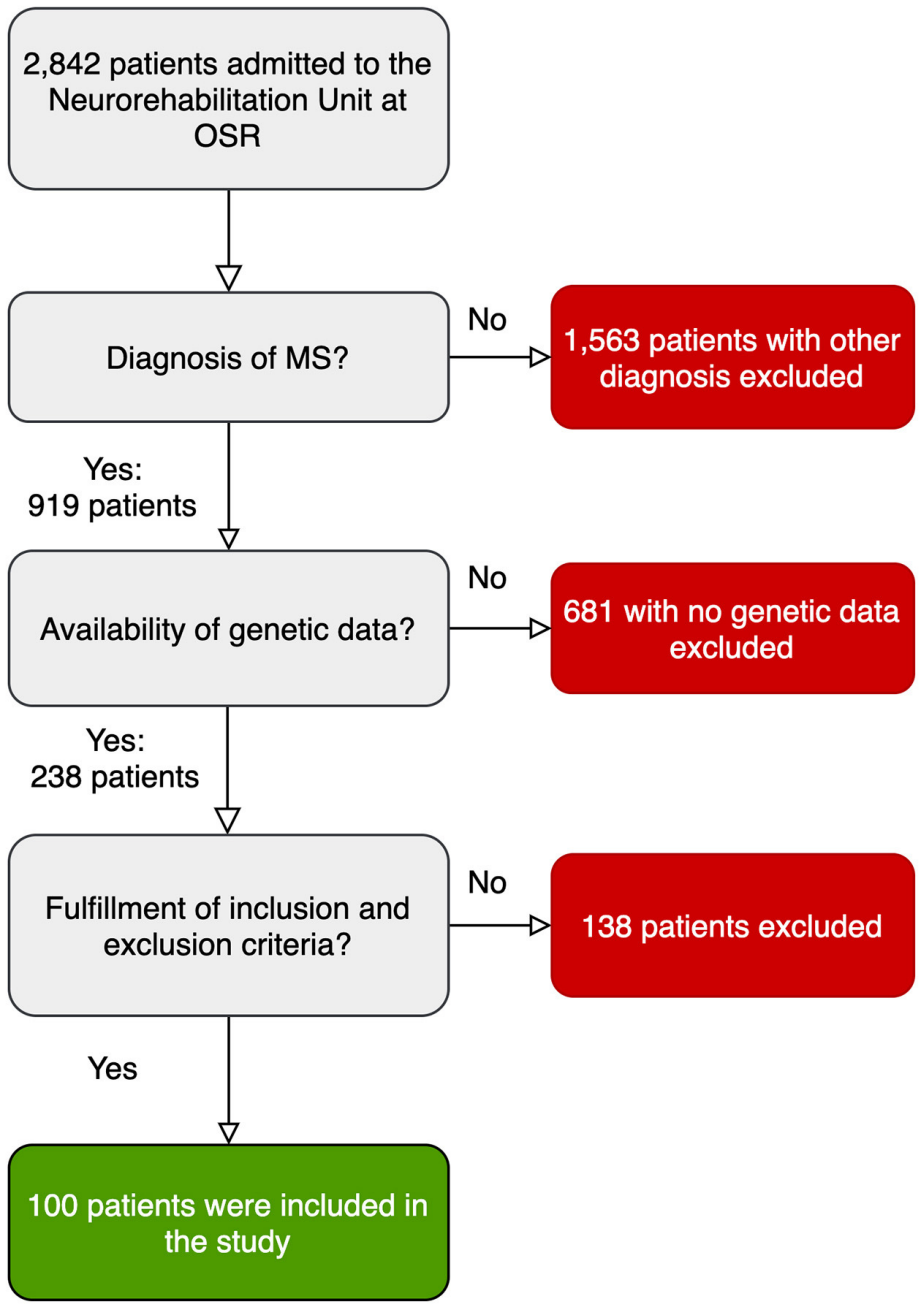
Flowchart showing the selection process for the included patients.

Between January 2011 and December 2018, 2,842 patients were admitted to the Neurorehabilitation Unity at OSR; among them, 919 were MS patients (Figure 1) and for 238 subjects whole genome genotyping data were available. After revision of medical records, 100 patients were included in the study according to the inclusion and exclusion criteria (Figure 1). Demographic and clinical characteristics of included patients are reported in Table 1. Among them, 89 subjects had data on 6MWT, 42 on 10MT and 46 patients had data on 9HPT.

**Table 1:** Clinical features of the included patients at baseline according to Val66Met BDNF genotype.

	**All**	**GG**	**AG/AA (Met-carriers)**	***p*-value**
Patients no.	100	68	32 (AG: 28, AA: 4)	-
Disease course	SP: 79; PP: 21	PP: 14; SP: 54	PP: 7; SP: 25	ns
Gender (F:M)	56:44	39:29	17:15	ns
Mean age	51.00 ± 10.10	50.80 ± 10.10	50.00 ± 10.40	ns
Mean EDSS	5.65	5.93	5.61	ns
EDSS range	3.5–7.0	3.5–7.0	3.5–7.0	-
6MWT, m (*n* = 89; 58 GG, 31 AG/AA)	199.10 ± 121.03	194.10 ± 127.30	208.70 ± 109.40	ns
10MT, s (*n* = 42; 31 GG, 11 AG/AA)	22.16 ± 18.39	24.97 ± 20.58	14.24 ± 4.72	ns
9HPT, s (*n* = 46; 31 GG, 15 AG/AA)	37.95 ± 31.42	57.71 ± 60.99	31.00 ± 10.15	ns

*6MWT, Six-minute walking test; 10MT, 10-Metre Test; 9HPT, Nine-Hole Peg Test; ns, non-significant. P-values were obtained using Chi-square test or Mann-Whitney test, when appropriate. For 6MWT, 10MT and 9HPT, the number of patients with available information, stratified also by genotype, is reported in brackets*.

In the selected cohort, the minor allele frequency of rs6265 was 0.18 and the Hardy-Weinberg Equilibrium was respected (*p* = 0.27). Specifically, 68 individuals were homozygous GG in rs6265, while 32 were carrier of at least one A allele (rs6265_AA_ and rs6265_AG_, representing the so-called Met-carriers). No significant differences in the clinical features were found at baseline evaluation according to *BDNF* Val66Met polymorphism before the rehabilitation program, pointing out that the two groups were comparable. Notably, also the results of motor functions tests (6MWT, 10MT and 9HPT) were comparable between the two genotype groups at baseline (Table 1).

As regards rs1212171 and rs2289656 in the *NTRK2* gene, 99 out 100 patients had available data on rs1212171 (TT: 32; TC 48; CC: 19), whereas 98 out of 100 subjects had available data on rs2289656 (CC: 54; CT: 34; TT: 10). Hardy-Weinberg Equilibrium was respected for these SNPs as well.

### Clinical and Demographic Predictors of Motor Recovery

As a preliminary analysis, we sought whether any clinical or demographic feature at baseline could predict the results of motor function tests after rehabilitation in univariable models (Supplementary Table 1). We found that higher EDSS at admission was associated with a worse outcome at 6MWT (*p* < 0.001) and 10MT (*p* = 0.006). In addition, a longer duration of the progressive phase was associated with worse motor recovery after rehabilitation treatment at 6MWT (*p* = 0.038), whereas male gender showed a slight negative relationship with 10MT results after rehabilitation (*p* = 0.048). Finally, lower 6MWT score at baseline (*p* = <0.001) or higher 10MT score at baseline (*p* = 0.002) were associated with worse results at 6MWT after rehabilitation training. Comparable results were obtained using 10MT change after rehabilitation as outcome both for baseline 6MWT (*p* = 0.002) and 10MT (*p* < 0.001). As regards 9HPT, no clinical or demographic variable predicted motor recovery after training, except for 9HPT result at baseline (*p* < 0.001).

### Effect of the Genetic Variants on Gait Function Recovery

**Figure 2:**
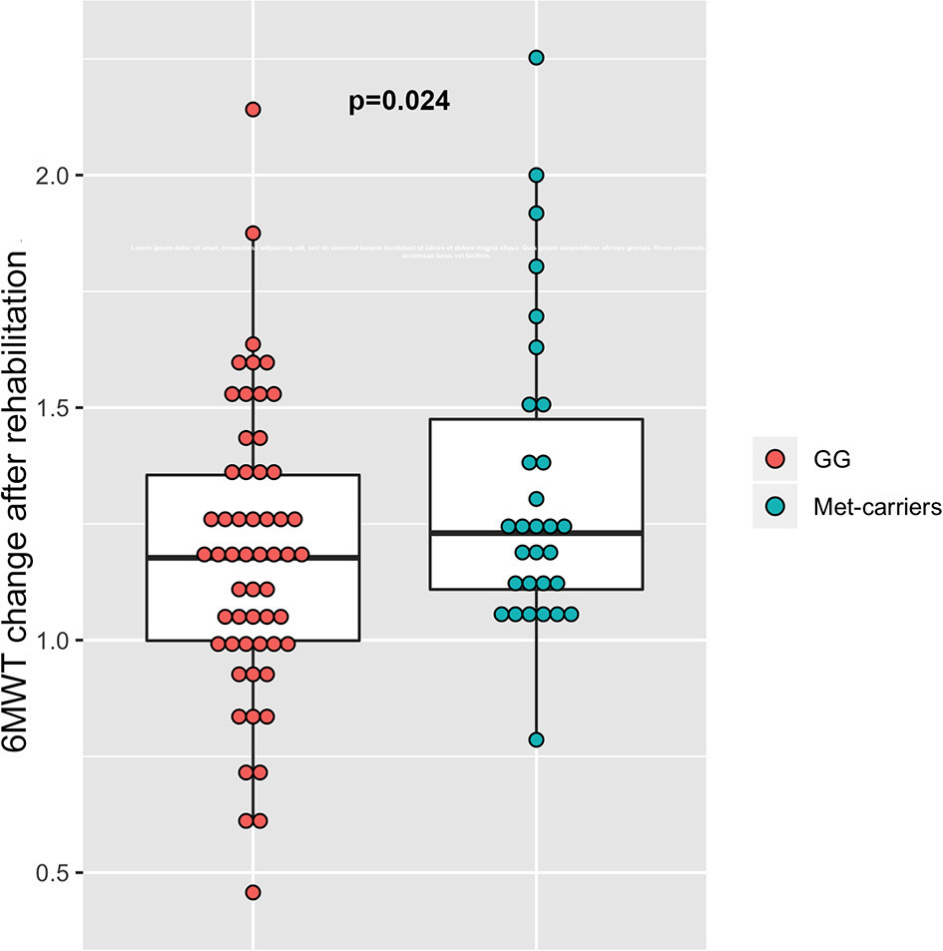
Association between rs6252 genotypes and results at 6MWT test. Dot-box plot shows that the change of 6MWT results is greater in Met-carriers (AG and AA subjects), compared to carriers of the more common GG genotype.

As shown in Table 2, considering the results of 6MWT test after rehabilitation as outcome of motor recovery, we found that *BDNF* Met-carriers (rs6265_A−carriers_) showed greater improvement after rehabilitation program, when compared to patients with the more common GG genotype (*p* = 0.024, beta = 0.16) (Figure 2). We did not find any significant association between the two SNPs in the *NTRK2* gene (rs2289656 and rs1212171) and the change of 6MWT results after rehabilitation, neither alone nor in interaction with the Val66Met polymorphism. When considering 10MT as outcome of the rehabilitation, no significant associations were found (Table 2).

**Table 2:** Association between the studied genetic variants of *BDNF* and *NTRK2* genes and the recovery of gait function after rehabilitation program.

	**Change of 6MWT (*****n*** **=** **89)**		**Change of 10MT (*****n*** **=** **42)**	
	**Beta (95% CI)**	***p*-value**	**Beta (95% CI)**	***p*-value**
*BDNF* rs6265_A	0.16 (0.02 to 0.29)	**0.024**	0.026 (−0.11 to 0.16)	ns
*NTRK2* rs2289656_T	−0.032 (−0.17 to 0.10)	ns	−0.001 (−0.19 to 0.035)	ns
*NTRK2* rs1212171_T	0.073 (−0.11 to 0.25)	ns	−0.0012 (−0.15 to 0.12)	ns
*BDNF* rs6265_A x *NTRK2* rs2289656_T	0.15 (−0.13 to 0.44)	ns	−0.043 (−0.32 to 0.23)	ns
*BDNF* rs6265_A x *NTRK2* rs1212171_T	0.23 (−0.19 to 0.64)	ns	−0.20 (−0.53 to 0.12)	ns

*6MWT, Six-Minutes Walking Tet; 10MT, 10-Metres Test; CI, confidence interval. Significant p-values reported in bold*.

### Effect of Genetic Variants on Hand Dexterity Recovery

When analyzing hand dexterity recovery by the change of 9HPT after rehabilitation, we did not find any significant association between the tested polymorphisms in *BDNF* or *NTRK2* genes and the change of the values of the test after rehabilitation.

## Discussion

The role of *BDNF* Val66Met polymorphism in neurological diseases has been explored in several studies in the past years.

Most of the existing literature focused on BDNF involvement in cognitive functions and the possible influence of the polymorphism on memory, executive functions and mood. In this field, an important work was published by Boots et al. (10), who found that episodic memory and executive functions impairment were more pronounced in *BDNF* Met-carriers in a large cohort of 1,023 subjects at risk for Alzheimer's disease, with the effect size being modulated by the burden of beta amyloid plaques. In another study conducted on 175 patients with Parkinson's disease, Met-carriers presented a higher prevalence of cognitive impairment (11).

When looking specifically at motor performances, very few papers have been published. In healthy subjects, the polymorphism was associated with altered short-term plasticity, greater error in short-term motor learning (8) and decreased training-induced amplitude of motor evoked potentials (9). Fischer et al. found that in a cohort of 217 patients with Parkinson's disease, carriers of two copies of the Met allele showed less severe motor symptoms and had a longer time from disease onset to the need for levodopa use, thus reflecting a possible slower progression in this group (31).

In the field of MS, data regarding *BDNF* Val66Met polymorphism are limited. The first studies investigated the possible role of this polymorphism as a risk factor for MS, but no association was found (14, 32). After these first works, scientific attention moved toward the evaluation of a potential correlation between the Val66Met polymorphism and gray matter atrophy. Overall, the data pointed out that the *BDNF* Met-carriers might benefit from a protective effect of the polymorphism against brain atrophy in MS. Indeed, despite one single work (15) showing that in 50 untreated RR MS patients gray matter volume was lower in Met-carriers, the results of all the subsequent research studies were in the opposite direction. In particular, Zivadinov et al. found that in Met-carriers gray matter volume was significantly higher and T2-lesion volume was lower in a cohort of 209 RR MS patients (16). Further evidence from the same and other groups confirmed that Met-carriers have higher gray matter volumes (18, 19) and hippocampal volumes (33).

In our study, *BDNF* Met-carriers showed greater improvement of the results of 6MWT after rehabilitation when compared to carriers of the more frequent GG genotype. These findings are in line with most of the existing studies that have analyzed the association between *BDNF* genotype and gray matter atrophy (16, 18, 19, 33). The biological link between gray matter atrophy and brain plasticity is well-known and it may represent one possible explanation for our results (34). We can speculate that the greater improvement of gait function after training observed in the Met-carriers group may reflect the minor degree of cortical atrophy, as described in previous studies (16, 18, 33). Of note, in these studies a different role of the Val66Met polymorphism was hypothesized for MS patients and healthy controls, as data seemed to point out an opposite effect in controls. The biological substrate of this phenomenon is not known, but the restoration of intracortical networks after neurological damage may be differently involved in MS patients and healthy controls. We hypothesize that the presence of the polymorphism could prevent the development of maladaptive cortical plasticity, thereby facilitating recovery. Evidence coming from studies on other neurological diseases, like ischemic stroke, seem to support this hypothesis (12).

Conversely, we did not find any significant effect of *BDNF* genotype when looking at 10MT as outcome of motor function recovery. This may be due to two pitfalls. First, the number of patients with this information was less than half compared to the ones with the data on 6MWT (42 *vs*. 89), which may have affected the results of the statistical analysis. Secondly, 10MT is a short-distance performance test, while 6MWT is a long-distance performance. According to previous studies, it has emerged that 6MWT may be the gold standard to test gait function in patients with MS (22), as it takes into account several factors which may influence this complex ability (e.g. fatigue, inter and intra-days variability, etc.).

Finally, we tried to explore the role of genetic variants in *NTRK2* gene on motor recovery. We did not find any significant effect of the studied SNPs, neither alone nor in association with Val66Met, with rehabilitation outcomes.

In a scenario where it is difficult to delineate clear pathways and biological mechanisms involved in motor recovery, our study tries to address some interesting points. First, we decided to focus on progressive MS patients with no evidence of recent clinical and/or neuroradiological inflammatory activity. Thanks to this approach, by eliminating the effect of possible inflammatory disease activity, we aimed to look more specifically at the neurodegenerative component of the disease, to better study its implications in motor learning. Another novelty is that we tried to investigate specifically motor learning and recovery, as this aspect was only partially addressed by previous works, despite its importance for MS patients. We also attempted to provide a more complete evaluation of the biological pathway involved in brain plasticity, assessing also genetic variants on the BDNF receptor, which role still needs to be clarified in MS.

On the contrary, the present work has also limitations, the most evident being the small sample size, which impacts on statistical power. Nevertheless, we detected an association between the *BDNF* rs6265 polymorphism and 6MWT change after rehabilitation, suggesting that there is an underlying relationship between BDNF and rehabilitation outcome, that is worth to test and replicate in a larger cohort. We cannot exclude that non-significant results observed in the present study are affected by the low statistical power, thus larger studies are necessary to confirm them. Another important limitation is the retrospective nature of the study. A prospective design would be preferable to minimize the risk of bias due to other external factors that may influence our outcome measures. However, both a sensitivity analysis and a comparison of clinical features at baseline across individuals with different genotypes did not reveal any significant issue for the explored factors, therefore partly addressing this problem. Of course, given the retrospective design, we could not take into account all the factors that may have a relevance in this phenomenon (e.g. fatigue, drugs that can impact on brain cortex excitability, etc.). In this context, prospective multicentric studies could help to enroll a larger number of patients and to control for potential biases, eventually assessing motor recovery not only by the results of clinical tests but also evaluating neurophysiological or functional MRI measures.

## Conclusions

In the present study we found that the Val66Met polymorphism is associated with the recovery of walking impairment after rehabilitation in people with progressive MS; these data are in line with previous studies suggesting the protective role of Val66Met polymorphism toward gray matter atrophy in MS. If confirmed in larger future studies, Val66Met polymorphism may represent a prognostic marker to better select patients who could benefit more from intensive neurorehabilitation programs.

## Data Availability Statement

The datasets for this article are not publicly available for ethical and legal reasons due to concerns regarding participant/patient anonymity. Requests to access the datasets should be directed to the corresponding author.

## Ethics Statement

The studies involving human participants were reviewed and approved by Ethical Committee, IRCCS San Raffaele Hospital, Milan (Italy). The patients/participants provided their written informed consent to participate in this study.

## Author Contributions

AG conceived the present idea, collected and analyzed the data, and wrote the manuscript. FC participated in the analysis of the data and drafting of the manuscript. MC participated in data collection and drafting of the manuscript. EM, SS, and MS carried out the genotyping experiments and contributed to manuscript drafting. LF, LL, and FE contributed to the conception and drafting of the manuscript. All authors contributed to the article and approved the submitted version.

## Conflict of Interest

LL reports personal fees for speaking or consulting activities from Roche, Merck, Bristol Myers Squibb, Med-ex learning, outside the submitted work. FE received compensation for consulting services and/or speaking activities from Novartis, Sanofi Genzyme, Almirall, Teva, and Merck-Serono. The remaining authors declare that the research was conducted in the absence of any commercial or financial relationships that could be construed as a potential conflict of interest.

## Publisher's Note

All claims expressed in this article are solely those of the authors and do not necessarily represent those of their affiliated organizations, or those of the publisher, the editors and the reviewers. Any product that may be evaluated in this article, or claim that may be made by its manufacturer, is not guaranteed or endorsed by the publisher.
