# Supplementary material for: BDNF Val66Met Polymorphism Is Associated With Motor Recovery After Rehabilitation in Progressive Multiple Sclerosis Patients
Source: Front Neurol. 2022 Feb 21;13:790360. doi: 10.3389/fneur.2022.790360 (PMC8899087; doi:10.3389/fneur.2022.790360)
Supplement: Supplementary file 1 [file Table_1.DOCX]

|  | **6MWT change after rehabilitation**  **n=85** | | **10MT change after rehabilitation**  **n=39** | | **9HPT change after rehabilitation**  **n=43** | |
| --- | --- | --- | --- | --- | --- | --- |
|  | **Beta (95% CI)** | **p-value** | **Beta (95% CI)** | **p-value** | **Beta (95% CI)** | **p-value** |
| **Age** | -0.51 (-3.31 to 2.29) | ns | -0.25 (-0.70 to 0.21) | ns | -0.057 (-1.53 to 1.42) | ns |
| **Gender (male)** | 27.75 (-28.78 to 84.28) | ns | -9.53 (-18.97 to -0.09) | **0.048** | 16.42 (-12.30 to 45.14) | ns |
| **Duration of disease progression** | -6.17 (-11.69 to -0.65) | **0.029** | -0.26 (-1.28 to 0.77) | ns | 0.85 (-1.81 to 3.51) | ns |
| **Hospitalization duration (days)** | -4.24 (-10.10 to 1.62) | ns | -0.35 (-1.73 to 1.02) | ns | 1.88 (1.05 to 4.81) | ns |
| **SP course** | 45.01 (-25.74 to 115.75) | ns | 5.20 (-7.004 to 17.40) | ns | 14.99 (-21.71 to 51.69) | ns |
| **Baseline EDSS** | -99.20 (-121.49 to -76.90) | **<0.001** | 8.26 (2.58 to 13.94) | **0.006** | 14.58 (-1.98 to 31.14) | ns |
| **Baseline 6MWT** | 0.91 (0.78 to 1.04) | **<0.001** | -0.06 (-0.098 to -0.024) | **0.002** | -0.018 (-0.12 to 0.088) | ns |
| **Baseline 10MT** | -5.31 (-8.556 to -2.07) | **0.002** | 0.77 (0.70 to 0.85) | **<0.001** | -0.1003 (-1.05 to 0.85) | ns |
| **Baseline 9HPT** | -0.28 (-1.39 to 0.82) | ns | -0.04 (-0.37 to 0.28) | ns | 0.81 (0.71 to 0.91) | **<0.001** |

**Supplementary Table 1.** Association between baseline clinical and demographic characteristics and motor recovery after intensive rehabilitation program. Univariate linear regression models were used; beta values with 95% Confidence Interval (CI) and p-values are reported.
